# Utilising Spent Tea Leaves Powder as Functional Ingredient to Enhance the Quality of Non-Gluten Shortbread Cookies

**DOI:** 10.3390/foods12071557

**Published:** 2023-04-06

**Authors:** Wee Yin Koh, Xiao Xian Lim, Thuan Chew Tan, Hasmadi Mamat, Rovina Kobun, Babak Rasti

**Affiliations:** 1 Functional Foods Research Group, Faculty of Food Science and Nutrition, Universiti Malaysia Sabah, Kota Kinabalu 88400, Malaysia; 2 Food Technology Division, School of Industrial Technology, Universiti Sains Malaysia, Minden 11800, Malaysia; 3 Renewable Biomass Transformation Cluster, School of Industrial Technology, Universiti Sains Malaysia, Minden 11800, Malaysia; 4 Australasian Nanoscience and Nanotechnology Initiative, 8054 Monash University, Clayton, VIC 3168, Australia

**Keywords:** green tea, oolong tea, black tea, polyphenols, dietary fibre

## Abstract

The increasing prevalence of gluten-related disorders has led to higher consumer demand for convenient, gluten-free bakery products with health-promoting properties. In this study, non-gluten shortbread cookies were incorporated with various kinds of spent (green, oolong, and black) tea leaves powder (STLP) at 8% *w*/*w*. Cookies with STLP had significantly higher (*p* < 0.05) moisture (2.18–2.35%), crude fibre (14.5–14.9%), total dietary fibre (22.38–22.59%), insoluble dietary fibre (15.32–15.83%), soluble dietary fibre (7.06–7.66%), and ash (1.9–2.0%) contents, but were significantly lower (*p* < 0.05) in carbohydrate (53.2–53.9%) and energy value (426.4–428.2 kcal) compared to control cookies (1.62%; 1.43%; 6.82%; 4.15%; 2.67%; 7.70%; 62.2%; and 457.8 kcal, respectively). The addition of STLP significantly enhanced (*p* < 0.05) the antioxidant properties of the cookies. Non-gluten shortbread cookies with spent green tea leaves powder (GTC) received the highest (*p* < 0.05) score for all sensory attributes, including overall acceptability. In addition, the shelf-life quality of the formulated cookie samples in terms of the moisture content, water activity, colour, texture, microbiology, and sensory properties was maintained (*p* > 0.05) for at least 22 days at 25 °C. STLP, which would have been previously thrown away, could be utilized as a potential functional ingredient to produce non-gluten shortbread cookies with enhanced nutritional, physicochemical, microbiological, sensory, and antioxidative properties.

## 1. Introduction

Cookies (or biscuits) are small, flat-baked products formulated with three main ingredients: flour, fat, and sugar. While bread is usually consumed as a staple food, cookies are widely consumed as snacks by all age groups all over the globe. Cookies have long been found to have good consumer acceptance owing to their nutrient availability, organoleptic properties, palatability, convenience (ready-to-eat), low cost, and comparatively longer shelf-life [1]. In particular, shortbread cookies are soft-type biscuits characterised by their relatively higher fat content (20–60%), and signature short and crumbly texture.

Traditionally, wheat flour is the main ingredient in cookies. However, the high gluten content in wheat flour could aggravate adverse health effects, including Celiac disease, wheat allergies, and non-Celiac gluten sensitivities, in certain consumers [1]. Today, gluten-related disorders have become a global health problem, whereby the global prevalence is estimated at ~5% [1,2]. Hence, the development of gluten-free cookies is necessary for those with gluten-related disorders. According to the Codex standard for gluten-free foods, a food designated as gluten-free can be made using one or more ingredients that do not contain wheat, rye, barley, and oats [3]. Rice flour is the most widely used flour in developing gluten-free bakery products. Nevertheless, rice flour does not supply an adequate amount of dietary fibre and nutritional value. Hence, numerous attempts have been conducted to produce non-gluten bakery products by utilising agro-industrial waste that is rich in nutrients and bioactive compounds, e.g., coffee extract residues [4], goji berry by-product [5], grape pomace [6], and hog plum bagasse [7].

Tea (*Camellia sinensis* L.) is the second-most drunk beverage in the world, next to drinking water. Depending upon the fermentation degree, tea can be categorised into green (unfermented), oolong (semi-fermented, 30–60 % oxidation), and black (fully fermented, 80–90 % oxidation) [8]. Tea leaves are rich in polyphenols and have been reported to possess health-promoting effects, including antioxidant, antiviral, antiallergic, anti-inflammatory, anticancer, cardiovascular, and neuroprotective properties [9,10]. Studies have shown that the polyphenols found in both black and green tea possess the ability to impede the reproduction of the adenovirus [11] and herpes simplex virus [12]. After one to two weeks of consumption, oolong tea (10 g/1000 mL per day, taken after three daily meals) was found to significantly (*p* < 0.05) reduce allergic reactions in patients with severe atopic dermatitis [13]. In addition, the consumption of green tea extracts (1000 mg, two capsules/day) for 12 weeks significantly reduced the disease activity in patients with systemic lupus erythematosus, an inflammatory autoimmune disease [14]. Green tea extract (EGCG, epigallocatechin-3-O-gallate, 44.9 mg/day for 4 weeks) treatment reduced the proliferation rate of tumour cells [15]. The visual recognition memory of adults (aged 16–34 years) with Down’s syndrome was also seen to improve with a 12-month treatment of green tea extract (EGCG, 9 mg/kg/day) and cognitive training [16]. Furthermore, high content of lignin, which functions as dietary fibre, has also been found in the leaves of tea [17]. 

According to the latest data from Statista, in the year 2020, the global production and consumption of tea reached 7.02 billion metric tons [18] and 6.3 billion kilograms [19], respectively. In response to the increasing tea consumption, the amount of spent tea leaves has also increased and has subsequently caused environmental pollution. Because spent tea leaves are said to contain similar components with amounts similar to new tea leaves, the disposal of spent tea leaves also represents a loss of valuable resources [20]. Studies have reported on the application of spent tea leaves in the area of animal feed [21] and agricultural compost [22]. However, there is a scarcity of research on the usage of spent tea leaves in the development of new food products [17,23]. Thus, this work illustrates a novel approach utilising STLP as an ingredient for non-gluten shortbread cookie preparation.

In the present study, various (green, oolong, and black teas) kinds of STLP were incorporated into non-gluten shortbread cookies with the objective to investigate the effect of STLP incorporation on the proximate composition, antioxidative, microbiological, and sensory characteristics, as well as the shelf-life stability, of non-gluten shortbread cookies.

## 2. Materials and Methods

### 2.1. Ingredients

Green, oolong and black tea leaves produced from the same cultivar of tea leaves (*Camellia sinensis* var. *assamica*) were bought from Kong Wooi Fong (KWF) Food Industries Sdn Bhd, Kuala Lumpur, Malaysia. Rice flour, maize flour, sugar, unsalted butter, eggs, and almond powder were bought from a local supermarket in Pulau Pinang, Malaysia.

### 2.2. Preparation of STLP

Tea leaves (100 g) were steeped in boiling water (1 L, 100 °C) and swirled with a magnetic stirrer on a hot plate (StableTemp, Cole-Parmer, Vernon Hills, IL, USA) at a rate of 5 min for 100× *g*. Subsequently, the tea was filtered through a gauze cloth to separate the spent tea leaves. After being dried in an oven (Memmert, UF-260, Schwabach, Germany) at 40 °C for 48 h, the dried spent tea leaves were ground (Orimas FFC 23, Agrowindo, Malang, Indonesia), sieved (125 µm, Endecotts Ltd., London, UK), sealed in aluminium-laminated film bags, and stored at 4 °C.

### 2.3. Preparation of Non-Gluten Shortbread Cookies with STLP

The non-gluten shortbread cookies were produced in accordance with the recipe specified in Table 1. The level of replacement of rice flour with STLP was selected based on a preliminary sensory acceptability test (result not shown) [24]. Among the replacement levels (2, 4, 6, 8, and 10 % *w*/*w*), the highest possible replacement of rice flour with STLP was 8 %.

**Figure 1:**
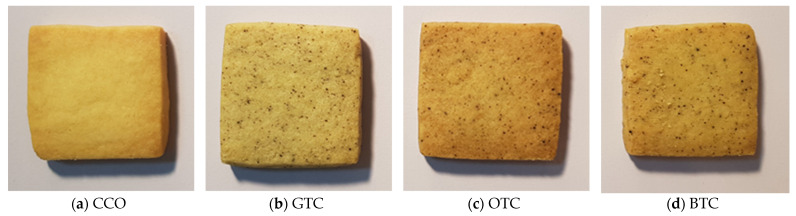
Non-gluten shortbread cookies with various spent tea leaf powders: (**a**) control non-gluten shortbread cookies without spent tea leaves powder (CCO), (**b**) non-gluten shortbread cookies with spent green tea leaves powder (GTC), (**c**) non-gluten shortbread cookies with spent oolong tea leaves powder (OTC), and (**d**) non-gluten shortbread cookies with spent black tea leaves powder (BTC).

The rice flour, maize flour, and STLP were mixed in a dough mixer (MK-HKM200, Panasonic, Osaka, Japan). The sugar, unsalted butter, precisely weighed egg yolk, and almond powder were added later into the dough and mixed thoroughly. The dough was then wrapped and covered with a plastic sheet. After being stored for 1 h in the refrigerator (4 °C, MC-4L1005, Midea Co., Ltd., Hefei, Anhui, China), the dough was rolled to 5 mm thickness, cut into squares (10 g, 3.5 × 3.5 cm), baked at 170 °C for 18 min in an oven (MBE-201SE-Z, Murni Bakery Equipment, Selangor, Malaysia), and left to cool at ambient temperature (25 °C) for 1 h. All the cookies (Figure 1) were sealed in airtight polyethylene bags, kept under ambient temperature (25 °C), and shielded against light.

### 2.4. Quality Properties of Non-Gluten Shortbread Cookies with STLP

#### 2.4.1. Proximate Composition

The proximate composition of the non-gluten shortbread cookies was conducted according to the Association of Official Analytical Chemists (AOAC) method [25]. The moisture, crude fat, total ash, crude protein, and crude fibre content of the cookies were determined through the oven drying method (AOAC 934.01; laboratory scale oven (UN-55, Memmert GmbH Co. KG, Schwabach, Germany), 105 °C), Soxhlet extraction (AOAC 930.09; solvent = diethyl ether (Sigma-Aldrich, St. Louis, MO, USA)), incineration method (AOAC 940.26; muffle furnace (Carbolite 1400, Carbolite Ltd., London, UK), 600 °C), Kjeldahl method (AOAC 979.09; protein-nitrogen conversion factor = 6.25), and fritted glass crucible method (AOAC 978.10). The insoluble (IDF) and soluble dietary fibre content (SDF) of the non-gluten shortbread cookies were determined by enzymatic-gravimetric methods (AOAC 991.42 and AOAC 993.19), whereas the total dietary fibre content (TDF) was calculated as the sum of IDF and SDF. The total carbohydrate and energy values were obtained based on percentage differences (Equation (1)) and conversion factors (Equation (2)).

Total carbohydrate (%) = 100% − (moisture + crude fat + ash + crude protein + crude fibre)%(1)

Energy value (kcal/100 g)                                              = [total carbohydrate (g/100 g) × 4 kcal/g] + [crude protein (g/100 g) × 4 kcal/g] + [crude fat (g/100 g) × 9 kcal/g](2)

#### 2.4.2. Determination of Tannin

The tannin content of the non-gluten shortbread cookies was determined according to the AOAC (AOAC 952.03) method [25]. First, cookies were crushed and dispersed in distilled water (1:10, *w*/*v*). After shaking for 30 min, the mixture was centrifuged and filtered to obtain the extract. Subsequently, 2 mL of cookie extract was mixed with 35 mL of distilled water, 1 mL of Folin–Denis reagent (Sigma-Aldrich, St. Louis, MO, USA), and 2.5 mL of sodium carbonate (35% *w*/*v*, Merck, Darmstadt, Germany). After diluting to 50 mL with distilled water, the mixture solution was incubated at room temperature (25 °C) for 90 min. The absorbance was then read at 760 nm using a spectrophotometer (Shimadzu, UV-160A PC, Kyoto, Japan).

#### 2.4.3. Characteristics of Cookie Doughs (Density and pH Value)

The density of the cookie dough was determined in accordance with the water displacement method described in [4]. About 3 g of the dough was weighed into a graduated cylinder partially filled with water. The volume of water displaced was recorded as the volume reading. The dough density was calculated as the ratio of the weight to the volume (g/mL). For the pH determination, 10 g of the dough was homogenised in 40 mL of distilled water. After being subjected to centrifugation (1000× *g*, 15 min), the pH value of the supernatant was measured.

#### 2.4.4. Characteristics of Cookies (Spread Factor, Loss Rate, Hardness, Colour, and Water Activity)

The spread factor (diameter/thickness) of the non-gluten shortbread cookies was measured and calculated according to the American Association of Cereal Chemistry (AACC) (10–50.05) [26]. The loss rate was determined by comparing the weight of the cookies before and after baking. The hardness of the cookies was measured by using a texture analyser (TA.XT2i Texture Analyzer, Stable Micro System, Goldaming, Surrey, UK) with a P5 cylindrical probe under the following operating conditions: compression (return to start), test speed of 1.5 mm/s, strain deformation of 80%, 20 mm limit, and trigger force and speed of 10 gf and 10 mm/min, respectively [4]. The colour values (CIE L*; lightness, CIE a*; redness, and CIE b*; yellowness) of the cookies were measured by using a reflectance spectrophotometer (CM-3500d, Konica Minolta Sensing, Inc., Osaka, Japan). The water activity of the cookies was measured by using a water activity meter (Aqualab, Pullman, WA, USA).

### 2.5. Antioxidative Properties of Non-Gluten Shortbread Cookies with STLP

The total phenolic (TPC) and flavonoid (TFC) contents, ferric-reducing antioxidant power (FRAP), and 2,2-diphenylpicryl hydrazine (DPPH) scavenging activity of the non-gluten shortbread cookies were determined according to the methods proposed by Rhee et al. [27], the National Food Research Institute [28], Blois [29], and Oyaizu [30], respectively, with slight modifications.

#### 2.5.1. Sample Preparations

The cookie samples were crushed and mixed with distilled water in a ratio of 1:10, agitated (60 min), centrifuged, and filtered to obtain the extracts. The resultant extract was concentrated to 1 mg/mL and kept in the refrigerator at −20 °C until used [4,31].

#### 2.5.2. Total Phenolic (TPC) and Flavonoid (TFC) Contents

To determine the TPC, 0.1 mL of cookie extract was mixed with 10 mL of 2% sodium bicarbonate (Merck, Darmstadt, Germany) and agitated for 2 min. After the addition of 0.1 mL of 50% Folin–Ciocalteu’s reagent (Sigma-Aldrich, St. Louis, MO, USA), the mixture was left in the dark for 30 min, and then the absorbance was taken at 750 nm using a spectrophotometer (Shimadzu, UV-160A PC, Kyoto, Japan). For the determination of TFC, 0.1 mL of cookie extract was incubated in a mixture of diethylene glycol (1 mL, Sigma-Aldrich, St. Louis, MO, USA) and 1 N sodium hydroxide (0.1 mL, Emsure, Merck KGaA, Darmstadt, Germany) for 60 min at 37 °C. The absorbance was read at 420 nm.

#### 2.5.3. Antioxidative Activities

The DPPH radical scavenging activity and FRAP assay were used to assess the antioxidant activities of the non-gluten shortbread cookies. To determine the DPPH radical scavenging activity, cookie extract (0.5 mL) was combined with 5 mL of a DPPH methanol solution (0.4 mM, HPLC grade, JT Baker, Phillipsburg, NJ, USA). After being left in the dark for 30 min, the absorbance of the mixture was measured at 517 nm. To perform the FRAP assay, 0.1 mL of cookie extract was dispersed in a mixture of 1% potassium ferricyanide (0.25 mL, Sigma-Aldrich, St. Louis, MO, USA) and 0.2 M phosphate buffer (0.25 mL, pH 6.6, Merck, Darmstadt, Germany), followed by incubation for 20 min at 50 °C. After the addition of 0.25 mL of 10% trichloroacetic acid (Bendosen Laboratory Chemicals, Bendosen, Norway), the mixture was centrifuged at 2500 rpm for 10 min. Subsequently, the supernatant (0.1 mL) was mixed with 0.1 mL of distilled water and 20 μL of 0.1% ferric chloride (Sigma-Aldrich, St. Louis, MO, USA), and the absorbance of the mixture was measured at 700 nm.

### 2.6. Microbiological Safety Analysis

The microbiological safety analysis of the non-gluten shortbread cookies was conducted prior to the hedonic sensory test. The cookie samples (25 g) were crushed under aseptic conditions and homogenised in sterile saline solution (225 mL, 0.85% *w*/*v*, HmbG Chemicals, Hamburg, Germany) for 30 min [32]. Subsequently, ten-fold serial dilutions were prepared. The dilutions of cookie samples were then plated on agar plates for enumeration. The total aerobic mesophilic count was estimated on Plate Count Agar (Merck, Frankfurt, Germany) using the pour plate technique (incubation: 30 °C, 72 h) [33]. The yeast and mould count were conducted on the Dichloran-Rose Bengal-Chloramphenicol agar (Merck, Frankfurt, Germany) using the spread plate technique (incubation: 25 °C, yeasts = 72 h, moulds = 120 h) [34,35]. The count of *Staphylococcus* spp. was carried out using the Baird Parker plates supplemented with Rabbit Plasma Fibrinogen (Sigma-Aldrich, St. Louis, MO, USA) with spread plate technique (incubation: 37 °C, 48 h) [36]. The detection of *Salmonella* spp. and *Escherichia coli* was performed on modified semi-solid Rappaport-Vassiliadis agar (Sigma-Aldrich, St. Louis, MO, USA) using the spread plate technique (incubation: 41.5 °C, 48 h) [37] and Tryptone Bile-glucuronide agar (Sigma-Aldrich, St. Louis, MO, USA) using the pour plate technique (incubation: 44 °C, 24 h) [38], respectively. Coliform bacteria were enumerated through pour plating on Violet Red Bile Agar (Sigma-Aldrich, St. Louis, MO, USA) (incubation: 30 °C, 48 h) [39]. For *Bacillus cereus*, the dilution was spread on a plate of Mannitol Egg Yolk Agar supplemented with polymyxin (Sigma-Aldrich, St. Louis, MO, USA) (incubation: 30 °C, 48 h) [40].

### 2.7. Sensory Analysis

The hedonic sensory test was conducted in a sensory evaluation lab according to the ISO 11136 standard [24]. An untrained group of 50 panellists (25 males, 25 females, between the ages of 20 and 60) was recruited to evaluate the sensory attributes (colour, smell, taste, texture (crumbly), and overall acceptability) of the non-gluten shortbread cookies using a seven-point hedonic scale (from “extremely like” to “extremely dislike”). Natural mineral water (Spritzer, Spritzer Bhd., Taiping, Malaysia) was served to rinse their mouth during the sensory test.

### 2.8. Storage Study

The storage stability study of the non-gluten shortbread cookies was carried out by sealing them in airtight polyethylene bags, storing them at ambient temperature (25 °C), and analysing them at an interval of 7 days for moisture content, water activity, colour, hardness, microbial load, and sensory attributes up to 22 days [41]. The moisture content of the cookies was determined using the oven drying method (AOAC 934.01) [25]. The water activity and colour of the cookies were measured by using a water activity meter (Aqualab, Pullman, WA, USA) and reflectance spectrophotometer (CM-3500d, Konica Minolta Sensing, Inc., Osaka, Japan), respectively. The hardness value was determined following the method described in Section 2.4.4 [4]. The microbial load (total aerobic mesophilic [33], yeasts and moulds [34,35], *Staphylococcus* spp. [36], *Salmonella* spp. [37], *Escherichia coli* [38], coliforms [39], and *Bacillus cereus* [40]) of the cookies during storage was determined according to methods proposed by the International Standard Organization (ISO). Sensory analysis was carried out according to the method mentioned in Section 2.7 [24].

### 2.9. Statistical Analysis

The significance of data was evaluated by a one-way analysis of variance and Duncan’s multiple range test at *p* < 0.05 using SPSS Statistics version 27 (IBM, Chicago, IL, USA) software. Results were shown as the mean ± SD from three independent parallel experiments. The Pearson test was used to study the correlation between TPC, TFC, and antioxidant activities (coefficients are significant at the significance level of 0.05).

## 3. Results and Discussion

### 3.1. Quality Properties of Non-Gluten Shortbread Cookies with STLP

#### 3.1.1. Proximate Composition

The nutritional composition of non-gluten shortbread cookies with and without the incorporation of STLP is presented in Table 2. In general, all the cookies formulated with STLP exhibited a significantly (*p* < 0.05) higher moisture, crude ash, and dietary fibre content, but significantly (*p* < 0.05) lower available carbohydrate content compared to the control cookies (CCO). There was no significant difference in the crude fat and protein content among the cookies with and without STLP.

Moisture content plays a vital role in the production of bakery goods, in which a lower moisture content could provide a crunchy structure and a longer shelf life to the cookies [7]. The moisture content of all the cookies formulated in this study ranged from 1.62 to 2.35%, which is in line with the findings that reported 1–5% moisture in cookies [19]. All the cookies with STLP had a significantly (*p* < 0.05) higher moisture content (2.18–2.35%) than that of the control (1.62%), indicating that the incorporation of STLP had significantly (*p* < 0.05) raised the moisture content of the cookies. The increase in the moisture content of GTC, OTC, and BTC could be due to the higher fibre content of STLP compared to rice flour. The cellulose in tea fibre readily interacts with water molecules through hydrogen bonding owing to their preponderance of hydroxyl functional groups. This allows the cookies to bind and keep more water, thus resulting in a higher moisture content [17]. From a food safety standpoint, the maximum allowed value of moisture for cookies recommended by the Food and Agriculture Organization/World Health Organization (FAO/WHO) is 4.5% [42]. The moisture content of all the cookies formulated in this study was lower than 4.5%, hence, they were within the acceptable moisture level and expected to be higher in microbiological durability and more stable against moisture-dependent biochemical and enzymatic deterioration.

The total ash content is a measure of the total amount of minerals present in a food. The total ash content of all the cookies was in the range of 1.43–2.04%. Significant (*p* < 0.05) increments were observed when STLP was added to the cookie formulation. The increase in the total ash content suggested that a significant amount of minerals was present in the STLP. Tea contains a rich amount of minerals, such as manganese, copper, magnesium, zinc, sodium, calcium, and iron [9].

The results of the crude fibre, total dietary fibre (TDF), insoluble dietary fibre (IDF), and soluble dietary fibre (SDF) analyses of the cookies are shown in Table 2. Significant differences (*p* < 0.05) were observed between the control (CCO; 7.7%) and cookies with STLP in terms of crude fibre content (14.5–14.9%). The cookies formulated with STLP also had a significantly higher TDF (22.38–22.59%), IDF (15.32–15.83%), and SDF (7.06–7.66%) compared to the control cookies (TDF: 6.82%; IDF: 4.15%; and SDF: 2.67%). The increase in fibre content was expected as spent tea leaves contain a high level of cellulose, lignin, hemicellulose, and crude fibre components [17]. According to the European Regulation (EC) No 1924/2006, a food product can be classified as “high in dietary fibre” with at least 6 g/100 g dietary fibre [43]. In the present study, all the cookies formulated can be considered food products with high dietary fibre. A good balance of IDF and SDF (ratio: 1.0 to 2.3 [44]) is essential for providing adequate nutritional effects and fibre functionality in the human diet. The IDF/SDF ratios of the control cookies and cookies with STLP were 1.55 and 2.07–2.17, respectively. Therefore, the dietary fibre proportion of all the cookie samples in the study was considered well-balanced.

Based on the results, the available carbohydrate content (53.2–53.9%) and energy value (426.4–428.2 kcal/100 g) of all the cookies with STLP were significantly (*p* < 0.05) lower than that of control (62.2% and 457.8 kcal/100 g, respectively). These findings suggested that the incorporation of STLP decreased the available carbohydrate content and energy value of cookies. The reduction in the available carbohydrate content and energy value makes STLP-incorporated cookies a potential candidate for health-promoting food when compared to the control cookies [10].

#### 3.1.2. Tannin Content

Tannin is a bioactive component associated with several health benefits but exhibits antinutritional properties when its value exceeds the acceptable safe level (10 mg/100 g) [45]. The tannin content of the non-gluten shortbread cookies with and without the incorporation of STLP is shown in Table 2. All the cookies formulated with STLP had a significantly (*p* < 0.05) higher tannin content than the control cookies (CCO). This might be attributed to the high tannin content of STLP. For instance, the tannin content in STLP is generally 3170–12,600 mg/100 g [46]. The tannin contents in cookies were in the order of BTC (6.10 mg/100 g) > OTC (4.21 mg/100 g) > GTC (2.33 mg/100 g) > CCO (0.28 mg/100 g). The tannin content of spent black tea powder was higher than that of spent oolong and green tea powders owing to the oxidation step involved in their production, which breaks down organic matter to produce tannins [47]. In the present study, the tannin contents of the cookie samples formulated in this study were considerably low. This could be attributed to the thermal degradation of tannin during the baking process [48]. Based on the results, the tannin content of all the cookies was within the acceptable range of its antinutritional effect on human consumption.

#### 3.1.3. Characteristics of Cookie Doughs (Density and pH Value)

The density of cookie dough is often measured to evaluate the quality of the final baked product. In general, dough with a lower density produces harder cookies with greater crispiness whereas dough with a higher density results in cookies with a weaker texture [4]. The dough density of all the cookie samples in this study ranged from 1.05 to 1.27 g/mL (Table 3), with no significant difference (*p* > 0.05) observed among the cookies. The incorporation of STLP neither affected the dough density nor the quality of the cookies.

The pH value of the cookie doughs varied from 7.15 to 7.75 (Table 3) and decreased (*p* < 0.05) upon the incorporation of STLP. This could be attributed to the slightly acidic nature of STLP [49]. Naturally, tea leaves contain a variety of acidic compounds such as caffeine and tannins.

#### 3.1.4. Characteristics of Cookies (Spread Factor, Loss Rate, Hardness, Colour, and Water Activity)

The effect of the partial replacement of rice flour with STLP on the spread factor, loss rate, and hardness of the cookies are shown in Table 4. The spread factor (width/thickness ratio) is a physical attribute that has long been used to evaluate the quality of cookies. In most cases, cookies with higher spread factors are known to have a better quality and higher consumer acceptability. Control cookies (CCO) were found to exhibit the lowest spread factor (4.58), followed by GTC (4.98), BTC (5.01), and OTC (5.36). The incorporation of STLP increased (*p* < 0.05) the spread factor of cookies, which could be due to the higher water-retaining capacity of the STLP-cookie dough (Table 2: moisture content = cookies with STLP > cookies without STLP). The stronger the ability of the dough to retain water, the lower the viscosity of the cookies. A lower dough viscosity could lead to a faster spreading rate during the baking, thus contributing to a higher spread factor for the cookies [50].

The weight loss of cookies is primarily due to the moisture loss from the dough during the baking process. According to the results, the loss rate of the cookies with STLP differed significantly (*p* < 0.05), with GTC (12.01%) demonstrating the lowest loss rate, followed by OTC (13.65%), and BTC (15.08%), which had the highest loss rate. The partial replacement of rice flour with spent green tea leaves powder significantly (*p* < 0.05) reduced the loss rate of cookies. However, an increase in loss rate was observed with spent black tea leaves powder.

The textural characteristic (hardness) is one of the most important criteria considered by consumers to evaluate the quality of a cookie. The cookie’s hardness corresponds to the force required to compress a cookie between the molars in the first bite until the cookie is cracked into two pieces. No significant difference (*p* > 0.05) in the hardness was found between the control cookies (3242.32 g) and cookies with STLP (3374.28–3398.64 g) (Table 4). According to Han and Lee [4], the hardness of cookies is greatly dependent on the protein content and the density of the cookie dough. In this study, the differences in protein content (Table 2) and dough density (Table 3) between the control cookies and cookies with STLP were not significant.

Colour is another important quality factor that affects the consumers’ overall acceptability of cookies. The appearance and colour of the cookies are presented in Figure 1 and Table 5, respectively. Compared to the control cookies, cookies with STLP had significantly (*p* < 0.05) lower CIE L*, b*, and a* values, indicating that the addition of STLP imparted darker colour to the cookies. These could be attributed to the original colour of STLP. STLP rich in polyphenols is darker in colour compared to white rice flour. During the production of tea, tea leaves undergo an enzymatic oxidation process catalysed by polyphenol oxidase. The oxidation process converts the colourless catechins in fresh tea leaves into theaflavins and thearubigins with a reddish-brown coloration, which later transforms the tea leaves to a dark brown colour [51]. Our observations are in agreement with Arı Akın et al. [17] who also found an increase in the darkness of bread upon the incorporation of tea fibre into the bread formulation.

Water activity is the measurement of the unbound water molecules in food and is one of the most important factors influencing the storage stability of dried foods. The United States Food and Drug Administration has established a 0.60 water activity threshold for preventing or limiting microbial growth in food [52]. Table 6 shows the water activities of the non-gluten shortbread cookies with various spent tea leaf powders. According to the results, cookies with STLP (0.22–0.24) had a significantly (*p* < 0.05) higher water activity compared to the control cookies (0.15). The increase in water activity after the addition of STLP was associated with the high moisture content of the STLP [17]. The water activity of the cookie samples in this study ranged from 0.15 to 0.24, which is significantly less than 0.6, thereby indicating the potential high storage stability of the cookies.

### 3.2. Antioxidative Properties of Non-Gluten Shortbread Cookies with STLP

#### 3.2.1. Total Phenolic (TPC) and Flavonoid (TFC) Contents

**Figure 2:**
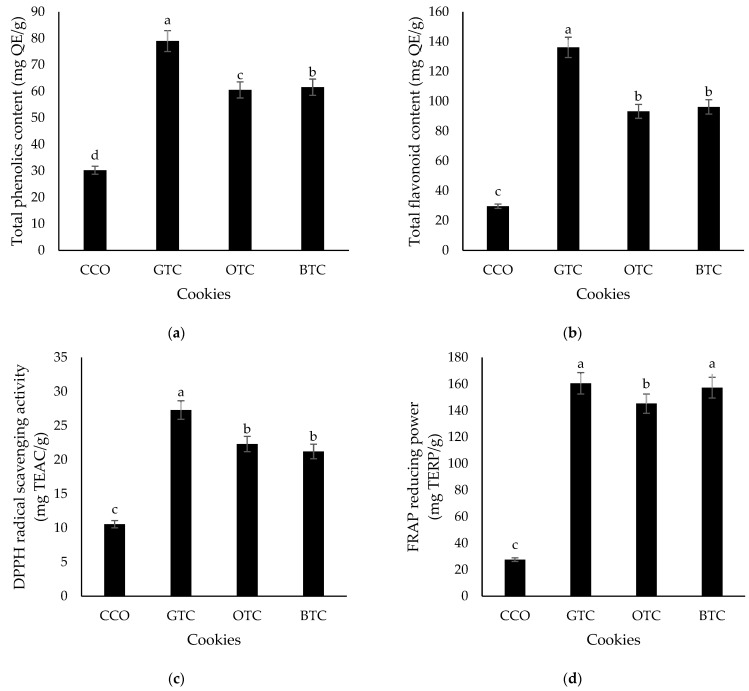
The antioxidative properties (**a**) total phenolics content, (**b**) the total flavonoid content, (**c**) DPPH radical scavenging activity, and (**d**) ferric reducing antioxidant power (FRAP) of non-gluten shortbread cookies with various spent tea leaf powders. The mean ± SD (n = 3). Different letters indicate significant differences between the values (Duncan’s multiple range test, *p* < 0.05, ^a–d^ cookie samples). CCO = control; GTC = non-gluten shortbread cookie with spent green tea leaves powder; OTC = non-gluten shortbread cookies with spent oolong tea leaves powder; and BTC = non-gluten shortbread cookies with spent black tea leaves powder.

In this study, the TPC and TFC of the cookies with and without STLP were measured by using water extraction and are presented in Figure 2a,b. The results showed that both the TPC and TFC of the control cookies (purely cereal-based) were significantly (*p* < 0.05) lower than those containing STLP. Tea is known to have rich phenolic compounds, especially catechin, tannin, theaflavin, and thearubigin [10]. When comparing the type of STLP used, cookies prepared from spent green tea leaves powder contained the highest TPC. The variation in the TPC and TFC of the cookies could be attributed to the extent of tea fermentation. In contrast to spent black and oolong tea leaf powders, spent green tea leaves powder retained more polyphenolic compounds as the fermentation (oxidation) step is bypassed in green tea production [53,54].

#### 3.2.2. Antioxidative Activities

The DPPH and FRAP values of all the cookies are shown in Figure 2c,d, respectively. The DPPH value of the control cookies (CCO) was significantly (*p* < 0.05) lower compared to the cookies containing STLP, implying that the incorporation of STLP had increased the antioxidant activities of cookies. STLP is rich in polyphenols with a great free radical scavenging ability. This result is in line with the TPC finding. Among the cookies with STLP, GTC had a significantly (*p* < 0.05) higher DPPH value than that of OTC and BTC. This indicated that spent green tea leaves powder was more effective in scavenging radicals and demonstrated a higher antioxidant activity compared to spent oolong and black tea leaf powders. The FRAP values of the cookies followed the same trend as TPC: GTC > BTC > OTC > CCO.

#### 3.2.3. The Relationship between TPC, TFC, and Antioxidative Activities

A high positive correlation was found between TPC and TFC (r = 0.999, *p* < 0.01). While TPC comprises both flavonoid and non-flavonoid phenolic compounds, the strong correlation indicated that flavonoids were the main phenolic compounds in cookies. The TPC and TFC of cookies showed high, positive correlations with DPPH (TPC: r = 0.991, *p* < 0.01; TFC: r = 0.853, *p* < 0.01) and FRAP (TPC: r = 0.986, *p* < 0.01; TFC: r = 0.872, *p* < 0.05). The results suggested that the phenolic compounds in the cookies were the predominant free radical scavenger and ferric reducer. FRAP was highly correlated (*p* < 0.01) with DPPH, with a correlation coefficient of 0.888. The high correlation of FRAP with DPPH suggested that the phenolic compounds present in the cookies could scavenge DPPH free radicals and reduce ferric ions.

### 3.3. Microbiological Quality

Microbiological testing was carried out to ensure the safety of cookie samples for human consumption. According to the maximum acceptable limits established by the FAO/WHO [42], at the time of consumption, the total count of aerobic mesophilic bacteria, yeasts and moulds, *Staphylococcus* spp., coliforms, and *Bacillus cereus* in baked products must not exceed 1 × 10^4^ CFU/g, 1 × 10^2^ CFU/g, 1 × 10 CFU/g, 1 × 10 CFU/g, and 1 × 10 CFU/g, respectively, with the absence of *Salmonella* spp. (0 CFU/25 g) and *Escherichia coli* (0 CFU/10 g). Based on the results obtained (Table 7), no growth of bacteria was observed in any of the cookie samples. The good microbial qualities of the cookies were attributed to the baking process. The temperature attained during baking was high enough to kill all viable microorganisms. Similar to our studies, no microbial growth was observed in the freshly baked edible insect powder-enriched cookies developed by Sriprablom et al. [55].

### 3.4. Sensory Analysis

**Figure 3:**
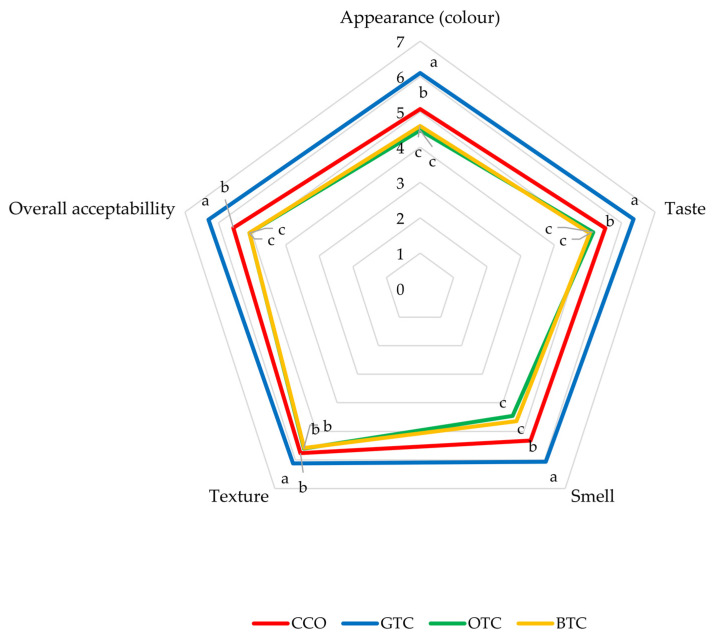
Sensory properties of non-gluten shortbread cookies with various spent tea leaf powders. The mean ± SD (*n* = 3). Different letters indicate significant differences between the values (Duncan’s multiple range test, *p* < 0.05, ^a–c^ cookie samples). CCO = control; GTC = non-gluten shortbread cookie with spent green tea leaves powder; OTC = non-gluten shortbread cookies with spent oolong tea leaves powder; BTC = non-gluten shortbread cookies with spent black tea leaves powder; 1 = extremely dislike; 2 = moderately dislike; 3 = slightly dislike; 4 = neither like nor dislike; 5 = slightly like; 6 = moderately like; and 7 = extremely like.

Sensory evaluation is the simplest and most direct method of testing the consumers’ acceptability of a food product. The sensory evaluation results of the non-gluten shortbread cookies are presented in a radar plot (Figure 3).

Based on the sensory results, GTC (non-gluten shortbread cookies with spent green tea leaves powder) were the best rated (*p* < 0.05) for all of the attributes. Among the four cookie samples, GTC scored the highest for its appearance. Spent green tea leaves powder gave GTC a light greenish colour that was desirable for some panellists. OTC and BTC were less acceptable due to their unappealing darker colour (Figure 1). Panellists preferred the texture, taste, and smell of GTC the most among the cookie samples. The partial replacement of rice flour with spent black and oolong tea leaves powder caused a significant negative effect on the texture, taste, and smell of the cookies. Most tea products possess a bitter aftertaste owing to the presence of catechins [56]. Panellists found no significant astringent aftertaste in GTC.

Overall acceptability is an important parameter in organoleptic estimation as it includes all the sensory attributes. Among all the cookies, panellists preferred GTC the most, followed by CCO, OTC, and BTC. Considering that GTC received the highest overall acceptance score, the partial replacement of rice flour with spent green tea leaves powder in the non-gluten shortbread cookie formulation clearly provided a better eating quality than the spent oolong and black tea leaf powders.

### 3.5. Storage Study

**Figure 4:**
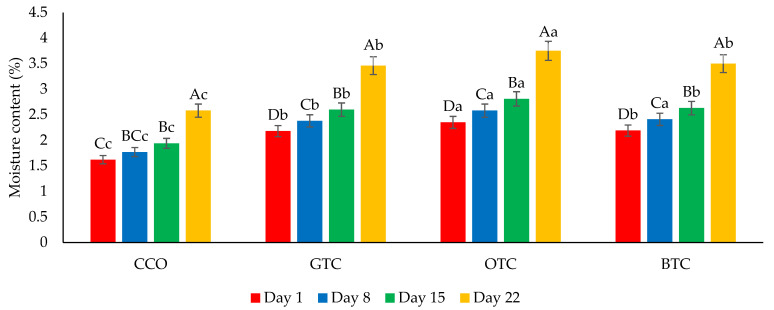
The moisture content of non-gluten shortbread cookies with various spent tea leaf powders during different storage periods (day 1, 8, 15, and 22) at 25 °C. The mean ± SD (*n* = 3). Different letters indicate significant differences between the values (Duncan’s multiple range test, *p* < 0.05, ^a–c^ cookie samples, ^A–D^ storage). CCO = control; GTC = non-gluten shortbread cookies with spent green tea leaves powder; OTC = non-gluten shortbread cookies with spent oolong tea leaves powder; and BTC = non-gluten shortbread cookies with spent black tea leaves powder.

**Figure 5:**
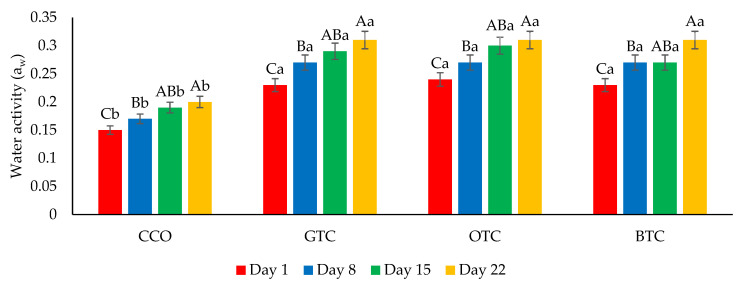
Water activity (a_w_) of non-gluten shortbread cookies with various spent tea leaf powders during different storage periods (day 1, 8, 15, and 22) at 25 °C. The mean ± SD (*n* = 3). Different letters indicate significant differences between the values (Duncan’s multiple range test, *p* < 0.05, ^a,b^ cookie samples, ^A–C^ storage). CCO = control; GTC = non-gluten shortbread cookies with spent green tea leaves powder; OTC = non-gluten shortbread cookies with spent oolong tea leaves powder; and BTC = non-gluten shortbread cookies with spent black tea leaves powder.

**Figure 6:**
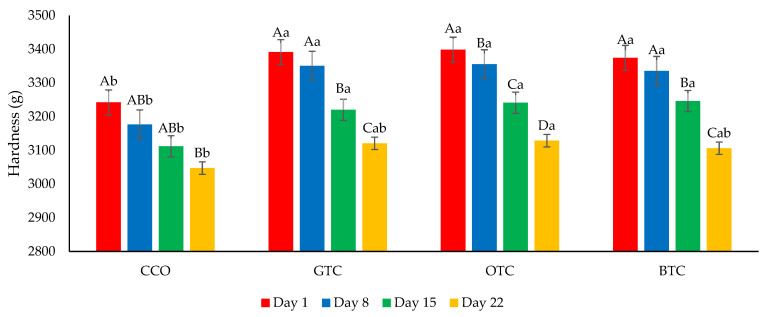
The hardness of non-gluten shortbread cookies with various spent tea leaf powders during different storage periods (day 1, 8, 15, and 22) at 25 °C. The mean ± SD (*n* = 3). Different letters indicate significant differences between the values (Duncan’s multiple range test, *p* < 0.05, ^a,b^ cookie samples, ^A–D^ storage). CCO = control; GTC = non-gluten shortbread cookies with spent green tea leaves powder; OTC = non-gluten shortbread cookies with spent oolong tea leaves powder; and BTC = non-gluten shortbread cookies with spent black tea leaves powder.

**Figure 7:**
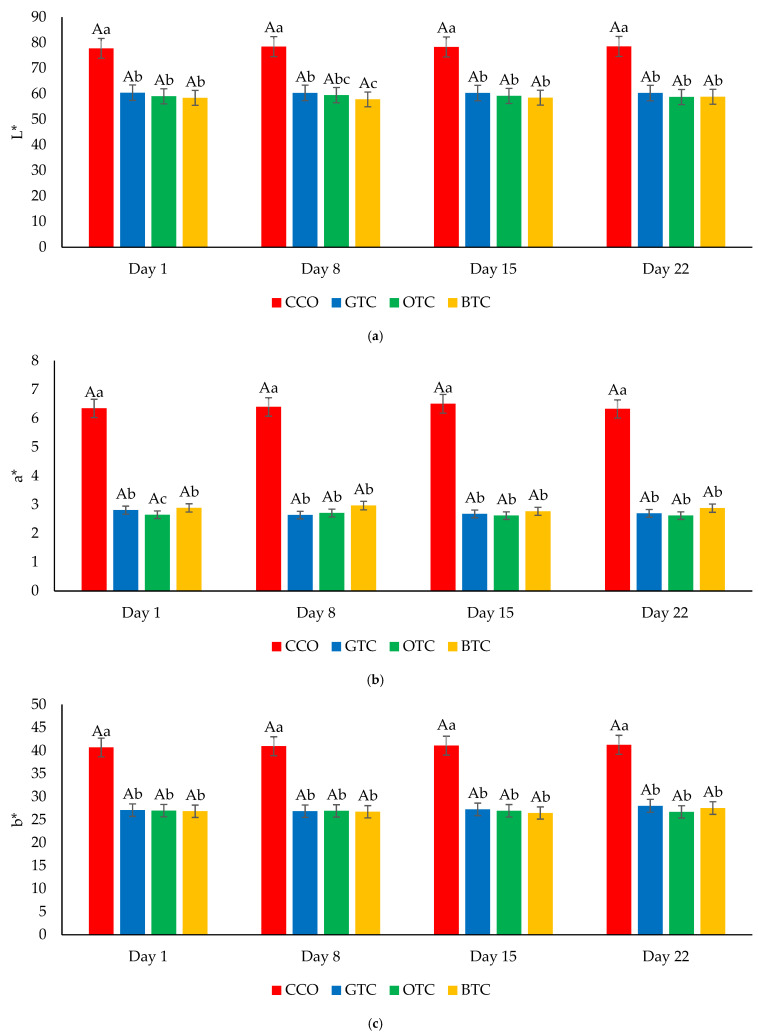
The colour (**a**) lightness, (**b**) redness, and (**c**) yellowness of non-gluten shortbread cookies with various spent tea leaf powders during different storage periods (day 1, 8, 15, and 22) at 25 °C. The mean ± SD (*n* = 3). Different letters indicate significant differences between the values (Duncan’s multiple range test, *p* < 0.05, ^a,b^ cookie samples, ^A^ storage). CCO = control; GTC = non-gluten shortbread cookies with spent green tea leaves powder; OTC = non-gluten shortbread cookies with spent oolong tea leaves powder; BTC = non-gluten shortbread cookies with spent black tea leaves powder; and L* lightness, a* redness, and b* yellowness.

The moisture content and water activity are critical factors in determining the quality and microbiological stability of dried food products such as cookies [7]. The changes in the moisture content and water activity in the cookie samples during storage are shown in Figure 4 and Figure 5, respectively. The moisture content and water activity of all the cookie samples significantly (*p* < 0.05) increased during the storage. The increase in moisture content in the cookie samples could be attributed to environmental moisture absorption, which corresponds to the hygroscopic nature of cookies. The increased water activity of the cookies was related to the increased moisture. Although the moisture content and water activity of all the cookie samples increased upon storage, the values were still lower than their respective maximum limiting values (moisture content = 4.5% [42]; water activity = 0.60 [52]) at the end of the storage period. The hardness of the cookies decreased significantly (*p* < 0.05) after being stored for 22 days (Figure 6). The decrease in the hardness of cookies during storage could be attributed to the increase in moisture content (Figure 4) and water activity (Figure 5). The ingress moisture softened the cookies [57] and the colour of the cookies was almost stable during 22 days of storage (Figure 7).

The growth of microorganisms is a critical factor in determining the shelf life of food products as microbiological deterioration remains the primary cause of food spoilage. The microbiological quality assessment of the non-gluten shortbread cookies carried out during the storage period is presented in Table 8. As per the results acquired, none of the cookie samples showed any visible signs of microbial growth until the 15th day of storage. On the 22nd day of storage, aerobic mesophilic bacteria were detected in all the cookies, with bacterial counts ranging from 2.23 × 10^2^ CFU/g to 2.68 × 10^2^ CFU/g, which is much lower than the permissible limit of 1 × 10^4^ CFU/g [42]. Based on the results, yeasts and moulds, *Staphylococcus* spp., *Salmonella* spp., coliforms, *Escherichia coli*, and *Bacillus cereus* were absent in all the cookie samples throughout the 22-day storage period. Based on the findings of this microbiological study, all of the cookie samples were deemed safe for consumption for a period of 22 days at 25 °C.

**Figure 8:**
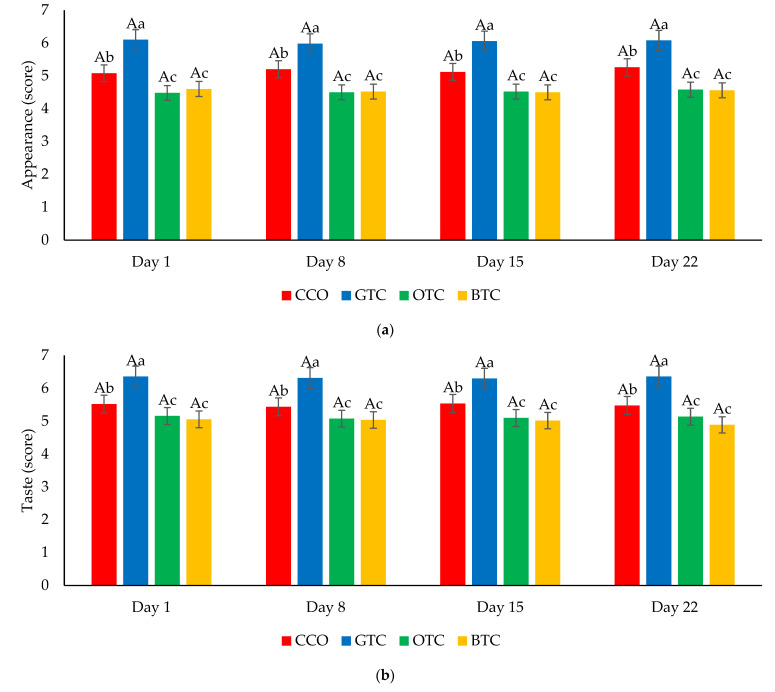

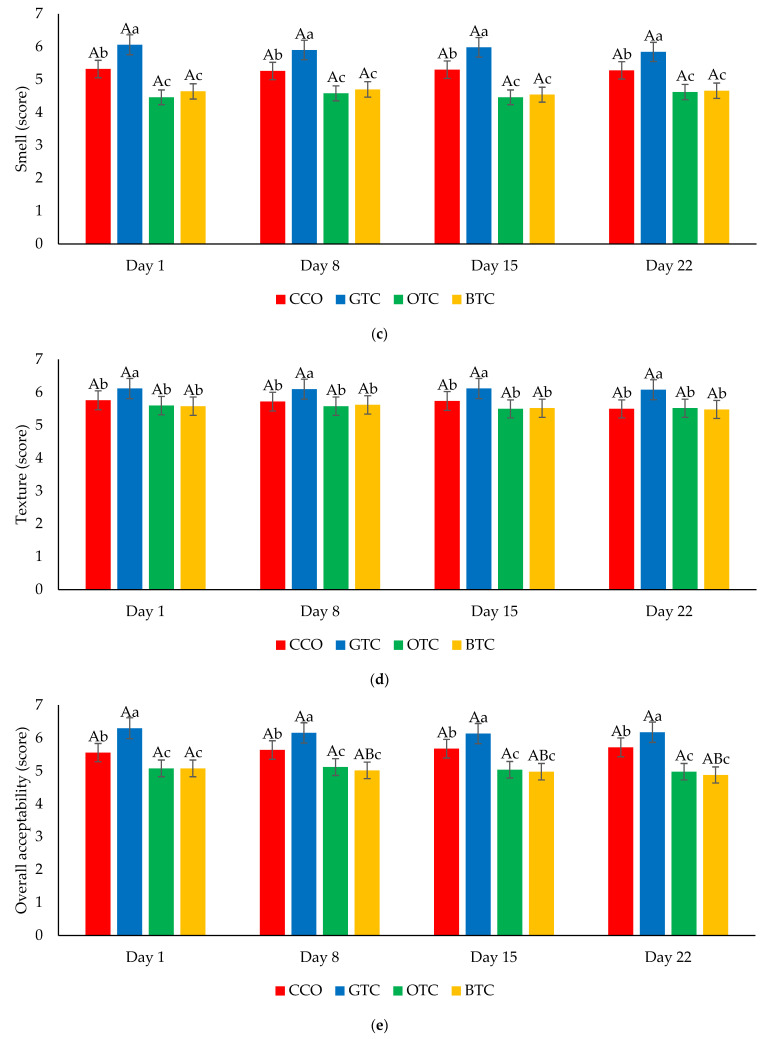
The sensory properties (**a**) appearance, (**b**) taste, (**c**) smell, (**d**) texture, and (**e**) overall acceptability of non-gluten shortbread cookies with various spent tea leaf powders during different storage periods (day 1, 8, 15, and 22) at 25 °C. The mean ± SD (*n* = 3). Different letters indicate significant differences between the values (Duncan’s multiple range test, *p* < 0.05, ^a–c^ cookie samples, ^A,B^ storage). CCO = control; GTC = non-gluten shortbread cookie with spent green tea leaves powder; OTC = non-gluten shortbread cookies with spent oolong tea leaves powder; BTC = non-gluten shortbread cookies with spent black tea leaves powder; 1 = extremely dislike; 2 = moderately dislike; 3 = slightly dislike; 4 = neither like nor dislike; 5 = slightly like; 6 = moderately like; and 7 = extremely like.

The sensory evaluation scores of the freshly baked and stored cookie samples are shown in Figure 8. The sensory scores of all the cookies were stable throughout the storage period of 22 days. The sensory panelists were unable to discern any differences between the freshly baked cookies and those that were stored for 22 days relative to all sensory attributes (*p* > 0.05). Results indicated that all the cookie samples retained good quality characteristics in terms of organoleptic properties until the end of the storage period.

## 4. Conclusions

Non-gluten shortbread cookies with spent green (GTC), oolong (OTC), and black (BTC) tea leaf powders were formulated and their nutritional composition, dough pH and density, physical, antioxidative, microbiological, and sensory characteristics, as well as the shelf-life stability, were investigated and compared with control cookies (CCO). The addition of STLP did not degrade the quality of the cookies and, in turn, enhanced their nutritional and antioxidant properties. Therefore, STLP, which would have been discarded in the past, is a potential, functional ingredient to be used in the production of non-gluten shortbread cookies. STLP could be used as a baking ingredient to develop gluten-free cookies with enhanced functional properties and achieve a sustainable food supply chain.

## Figures and Tables

**Table 1:** Formulation of non-gluten shortbread cookies with various spent tea leaf powders.

Ingredients (*% w*/*w*)	Cookies			
	CCO	GTC	OTC	BTC
Rice flour	32	24	24	24
Maize flour	15	15	15	15
Spent tea leaves powder	0	8	8	8
Unsalted butter	2.5	2.5	2.5	2.5
Sugar	24	24	24	24
Egg yolk	11.5	11.5	11.5	11.5
Almond powder	15	15	15	15

CCO = control; GTC = non-gluten shortbread cookies with spent green tea leaves powder; OTC = non-gluten shortbread cookies with spent oolong tea leaves powder; BTC = non-gluten shortbread cookies with spent black tea leaves powder.

**Table 2:** Proximate composition, energy value, and tannin content of non-gluten shortbread cookies with various spent tea leaf powders.

Component	Cookies			
	CCO	GTC	OTC	BTC
Moisture (%)	1.62 ± 0.24 ^c^	2.18 ± 0.03 ^b^	2.35 ± 0.01 ^a^	2.19 ± 0.01 ^b^
Crude fat (%)	20.17 ± 0.03 ^a^	20.60 ± 0.55 ^a^	20.80 ± 0.38 ^a^	20.39 ± 0.46 ^a^
Total ash (%)	1.43 ± 0.04 ^c^	2.04 ± 0.04 ^a^	1.91 ± 0.06 ^b^	1.96 ± 0.04 ^b^
Crude protein (%)	6.86 ± 0.10 ^a^	6.88 ± 0.04 ^a^	6.89 ± 0.10 ^a^	6.84 ± 0.05 ^a^
Crude fibre (%)	7.70 ± 0.04 ^b^	14.47 ± 0.45 ^a^	14.89 ± 0.09 ^a^	14.75 ± 0.06 ^a^
Total dietary fibre (%)	6.82 ± 1.35 ^b^	22.38 ± 1.07 ^a^	22.59 ± 1.28 ^a^	22.48 ± 1.16 ^a^
Insoluble dietary fibre (%)	4.15 ± 0.62 ^b^	15.32 ± 0.78 ^a^	15.44 ± 0.99 ^a^	15.83 ± 1.63 ^a^
Soluble dietary fibre (%)	2.67 ± 0.79 ^b^	7.06 ± 0.38 ^a^	7.15 ± 0.35 ^a^	7.66 ± 0.50 ^a^
Total carbohydrate (%)	62.21 ± 0.68 ^a^	53.83 ± 0.80 ^b^	53.16 ± 0.48 ^b^	53.87 ± 0.48 ^b^
Energy (kcal/100 g)	457.84 ± 0.71 ^a^	428.21 ± 2.68 ^b^	427.36 ± 1.80 ^b^	426.37 ± 2.44 ^b^
Tannin (mg/100 g)	0.28 ± 0.31 ^d^	2.33 ± 0.27 ^c^	4.22 ± 0.79 ^b^	6.10 ± 0.26 ^a^

The mean ± SD (*n* = 3). Different letters indicate significant differences between the values (Duncan’s multiple range test, *p* < 0.05, ^a–d^ cookie samples). CCO = control; GTC = non-gluten shortbread cookies with spent green tea leaves powder; OTC = non-gluten shortbread cookies with spent oolong tea leaves powder; and BTC = non-gluten shortbread cookies with spent black tea leaves powder.

**Table 3:** The density and pH of the dough of non-gluten shortbread cookies with various spent tea leaf powders.

Cookies	Density (g/mL)	pH
CCO	1.27 ± 0.05 ^a^	7.75 ± 0.03 ^a^
GTC	1.08 ± 0.08 ^a^	7.15 ± 0.01 ^c^
OTC	1.07 ± 0.06 ^a^	7.16 ± 0.01 ^c^
BTC	1.05 ± 0.09 ^a^	7.38 ± 0.02 ^b^

The mean ± SD (*n* = 3). Different letters indicate significant differences between the values (Duncan’s multiple range test, *p* < 0.05, ^a–c^ cookie samples). CCO = control; GTC = non-gluten shortbread cookies with spent green tea leaves powder; OTC = non-gluten shortbread cookies with spent oolong tea leaves powder; and BTC = non-gluten shortbread cookies with spent black tea leaves powder.

**Table 4:** Spread factor, loss rate, and hardness of non-gluten shortbread cookies with various spent tea leaf powders.

Cookies	Texture		
	Spread Factor	Loss Rate (%)	Hardness (g)
CCO	4.58 ± 0.02 ^c^	13.76 ± 0.05 ^b^	3242.32 ± 384.18 ^a^
GTC	4.98 ± 0.01 ^b^	12.01 ± 0.04 ^c^	3391.29 ± 359.72 ^a^
OTC	5.36 ± 0.02 ^a^	13.65 ± 0.07 ^b^	3398.64 ± 142.54 ^a^
BTC	5.01 ± 0.03 ^b^	15.08 ± 0.04 ^a^	3374.28 ± 531.18 ^a^

The mean ± SD (*n* = 3). Different letters indicate significant differences between the values (Duncan’s multiple range test, *p* < 0.05, ^a–c^ cookie samples). CCO = control; GTC = non-gluten shortbread cookies with spent green tea leaves powder; OTC = non-gluten shortbread cookies with spent oolong tea leaves powder; and BTC = non-gluten shortbread cookies with spent black tea leaves powder.

**Table 5:** Colours of non-gluten shortbread cookies with various spent tea leaves powder.

Cookies	Colour Parameters		
	L*	a*	b*
CCO	77.78 ± 0.65 ^a^	6.35 ± 0.48 ^a^	40.71 ± 0.92 ^a^
GTC	60.43 ± 1.12 ^b^	2.81 ± 0.15 ^b^	27.08 ± 1.36 ^b^
OTC	59.03 ± 0.98 ^b^	2.65 ± 0.29 ^c^	26.98 ± 0.22 ^b^
BTC	58.41 ± 1.03 ^b^	2.89 ± 0.21 ^b^	26.84 ± 0.74 ^b^

The mean ± SD (*n* = 3). Different letters indicate significant differences between the values (Duncan’s multiple range test, *p* < 0.05, ^a–c^ cookie samples). CCO = control; GTC = non-gluten shortbread cookies with spent green tea leaves powder; OTC = non-gluten shortbread cookies with spent oolong tea leaves powder; BTC = non-gluten shortbread cookies with spent black tea leaves powder; and L* lightness, a* redness, and b* yellowness.

**Table 6:** The water activity of non-gluten shortbread cookies with various spent tea leaf powders.

Cookies	Water Activity
CCO	0.15 ± 0.01 ^b^
GTC	0.23 ± 0.02 ^a^
OTC	0.24 ± 0.02 ^a^
BTC	0.22 ± 0.02 ^a^

The mean ± SD (*n* = 3). Different letters indicate significant differences between the values (Duncan’s multiple range test, *p* < 0.05, ^a,b^ cookie samples). CCO = control; GTC = non-gluten shortbread cookies with spent green tea leaves powder; OTC = non-gluten shortbread cookies with spent oolong tea leaves powder; and BTC = non-gluten shortbread cookies with spent black tea leaves powder.

**Table 7:** Microbiological quality of non-gluten shortbread cookies with various spent tea leaf powders.

Microbial Load (CFU/g)	Reference Value (Maximum Level) *	Cookies			
		CCO	GTC	OTC	BTC
Total aerobic mesophilic	1 × 10^4^ CFU/g	<10	<10	<10	<10
Yeasts and moulds	1 × 10^2^ CFU/g	<10	<10	<10	<10
*Staphylococcus* spp.	1 × 10 CFU/g	<10	<10	<10	<10
*Salmonella* spp.	Absent in 25 g	Absent	Absent	Absent	Absent
Total coliform	1 × 10 CFU/g	<10	<10	<10	<10
*Escherichia coli*	Absent in 10 g	Absent	Absent	Absent	Absent
*Bacillus cereus*	1 × 10 CFU/g	<10	<10	<10	<10

The mean ± SD (*n* = 3). CCO = control; GTC = non-gluten shortbread cookies with spent green tea leaves powder; OTC = non-gluten shortbread cookies with spent oolong tea leaves powder; BTC = non-gluten shortbread cookies with spent black tea leaves powder; and < 10 = absence of test microorganisms in 1 g of sample. * = reference value from FAO/WHO [42].

**Table 8:** The microbiological quality of non-gluten shortbread cookies with various spent tea leaf powders during different storage periods (day 1, 8, 15, and 22) at 25 °C.

Microbial Load (CFU/g)	Reference Value (Maximum Level) *	Days	Cookies			
			CCO	GTC	OTC	BTC
Total aerobic mesophilic	1 × 10^4^ CFU/g	1	<10	<10	<10	<10
		8	<10	<10	<10	<10
		15	<10	<10	<10	<10
		22	2.41 × 10^2^	2.68 × 10^2^	2.23 × 10^2^	2.55 × 10^2^
Yeasts and moulds	1 × 10^2^ CFU/g	1	<10	<10	<10	<10
		8	<10	<10	<10	<10
		15	<10	<10	<10	<10
		22	<10	<10	<10	<10
*Staphylococcus* spp.	1 × 10 CFU/g	1	<10	<10	<10	<10
		8	<10	<10	<10	<10
		15	<10	<10	<10	<10
		22	<10	<10	<10	<10
*Salmonella*	Absent in 25 g	1	Absent	Absent	Absent	Absent
		8	Absent	Absent	Absent	Absent
		15	Absent	Absent	Absent	Absent
		22	Absent	Absent	Absent	Absent
Total coliform	1 × 10 CFU/g	1	<10	<10	<10	<10
		8	<10	<10	<10	<10
		15	<10	<10	<10	<10
		22	<10	<10	<10	<10
*Escherichia coli*	Absent in 10 g	1	Absent	Absent	Absent	Absent
		8	Absent	Absent	Absent	Absent
		15	Absent	Absent	Absent	Absent
		22	Absent	Absent	Absent	Absent
*Bacillus cereus*	1 × 10 CFU/g	1	<10	<10	<10	<10
		8	<10	<10	<10	<10
		15	<10	<10	<10	<10
		22	<10	<10	<10	<10

The mean ± SD (*n* = 3). CCO = control; GTC = non-gluten shortbread cookies with spent green tea leaves powder; OTC = non-gluten shortbread cookies with spent oolong tea leaves powder; BTC = non-gluten shortbread cookies with spent black tea leaves powder; and < 10 = absence of test microorganisms in 1 g of sample. * = reference value from FAO/WHO [42].

## Data Availability

Data are contained within the article.
